# Prevalence and factors associated with social media addiction among undergraduate students from selected districts of Kerala, India: a cross-sectional study

**DOI:** 10.1186/s12889-026-28841-9

**Published:** 2026-08-03

**Authors:** Swathy Krishna, Jeby Jose Olickal, Kavumpurathu Raman Thankappan

**Affiliations:** 1 Department of Public Health, Amrita School of Medicine, Amrita Vishwa Vidyapeetham, Kochi, Kerala India; 2 Department of Community Medicine and School of Public Health, Postgraduate Institute of Medical Education and Research (PGIMER), Chandigarh, India

**Keywords:** Social Media Addiction, Behavioural Addiction, Young Adults

## Abstract

**Background:**

Although evidence on Social Media Addiction (SMA) is growing globally, research from India remains limited. This study aimed to estimate the prevalence of SMA and its associated factors among undergraduate students from selected districts of Kerala, India.

**Methods:**

This cross-sectional study included 823 Arts & Science and Engineering college students (mean age 19.8 years, 60% female) from two districts in Kerala, selected through a multistage cluster sampling design. The Bergen Social Media Addiction Scale (BSMAS) was used to assess SMA. Survey-weighted generalized linear models with a Poisson family and log link were used to identify factors associated with SMA.

**Results:**

The survey-weighted prevalence of SMA was 38.07% (95% CI: 33.56–42.81), while the prevalence of social media disorder (SMD) was 4.03% (95% CI: 2.69–5.99). Among participants with SMA, 12.67% met the criteria for SMD. In the adjusted analysis, female students had a higher prevalence of SMA than males (APR = 1.42; 95% CI: 1.07–1.87; p = 0.018). Married students had a higher prevalence of SMA than unmarried students (APR = 1.73; 95% CI: 1.33–2.24; p < 0.001), although the number of married participants was small. Students who used social media for more than 5 h per day had a higher prevalence of SMA than those using it for 0–5 h per day (APR = 1.41; 95% CI: 1.12–1.78; p = 0.006). A weak positive correlation was observed between daily social media duration and BSMAS score (Spearman’s rho = 0.183, p < 0.001).

**Conclusion:**

In this study of 823 undergraduate students, more than one-third were affected by SMA, with a notable proportion experiencing SMD. These findings highlight the need for targeted digital well-being interventions in college settings that focus on reducing excessive screen time and promoting responsible social media use.

## Introduction

Social media addiction (SMA) is a global public health concern, yet defining and measuring SMA remains challenging due to its multi-dimensional nature [1]. SMA is typically characterised by excessive and compulsive use of social media platforms, leading to significant impairment in daily functioning, academic performance, and interpersonal relationships [2]. SMA is usually assessed using the Bergen Social Media Addiction Scale (BSMAS), the most widely used measure of SMA, a validated six-item self-report instrument developed based on the core components of addiction: salience, mood modification, tolerance, withdrawal, conflict, and relapse [3]. The BSMAS has been widely applied across diverse populations and demonstrates strong psychometric properties, making it a suitable tool for screening for SMA among young adults [4, 5]. While internet addiction encompasses a broad range of problematic online behaviours, including gaming, browsing, and streaming, SMA specifically refers to compulsive and excessive engagement with social networking platforms. Young adults are particularly vulnerable as they rapidly adapt to digital technologies, and research suggests a strong correlation between internet addiction and SMA. Contemporary behavioural addiction frameworks suggest that problematic online behaviours arise through interactions between individual characteristics, emotional factors, cognitive processes, and patterns of media use [6].

Social media usage has increased significantly, with the global average reaching 151 min per day in 2023 [7]. A systematic review by Tadpatrikar et al. reported a pooled prevalence of 58.34% for internet addiction in Indian adults [8]. In comparison, Kumar T et al. found a 53.6% prevalence of internet addiction among individuals aged 18 years or older [9]. Social Networking Sites (SNSs) like Instagram, YouTube, Facebook, Twitter, and WhatsApp facilitate online interactions [10]. A meta-analysis by Cheng et al. reported that SMA prevalence was higher in Africa (37%), Asia (31%), and the Middle East (29%) than in North America (15%) and Western/Northern Europe (8%) [11].

While studies have examined internet addiction in India, research specifically focusing on SMA remains limited. Ramesh et al. (2018) reported a 36.9% prevalence of SMA among adolescents in urban Bengaluru using a non-standardised scale [12]. Still, evidence on its burden and associated factors among undergraduate students and young adults in India remains scarce. This population represents a potentially high-risk group due to frequent and prolonged engagement with digital platforms for academic, social, and recreational purposes. Furthermore, there is a paucity of data from Kerala, a state with one of the highest levels of mobile phone penetration and internet usage in the country, with 76.2% of women and 76% of men reporting internet use [13]. Given this unique socio-digital context, understanding SMA in this population is important. In addition to commonly studied factors such as gender and screen time, certain demographic and lifestyle characteristics, including body mass index (BMI), and family structure, have been examined in relation to behavioural and mental health outcomes, although evidence remains limited and inconsistent. Therefore, these variables were included as exploratory covariates in the present study rather than as a hypothesis-driven primary associated factor. This study aimed to determine the prevalence of SMA and its associated factors among undergraduate students aged 18 to 22 years from selected districts of Kerala.

## Methods

### Study design

The present study is a secondary analysis of data collected as part of a larger cross-sectional study conducted among undergraduate students from selected districts of Kerala. The parent study primarily investigated eating disorder risk among the same study population, and its findings have been published elsewhere [14].

### Study population

This study recruited undergraduate students aged 18–22 years enrolled in Arts & Science and Engineering courses at colleges in Kollam and Kozhikode districts of Kerala, India.

### Study duration

The study spanned eight months, from November 2023 to May 2024. The data collection period was from February 15^th,^ 2024, to April 15^th,^ 2024.

### Inclusion and exclusion criteria

Undergraduate students aged 18–22 years enrolled in Arts & Science and Engineering colleges in the selected districts were eligible for inclusion in the study. Students who provided written informed consent and were present in the selected classrooms at the time of data collection were included.

Students who did not provide consent or had incomplete responses to the BSMAS were excluded from the analysis.

### Sample size

The sample size was calculated as part of a larger study assessing the risk of eating disorders among college students. The required sample size was estimated using the formula for a single proportion, assuming a prevalence of 26.06% based on previous literature [15], with 5% absolute precision and a 95% confidence level. A design effect of 2 was applied to account for the cluster sampling design, resulting in a minimum sample size of 592. After accounting for an anticipated non-response rate of 20%, the final sample size was estimated at 740 participants. However, all eligible students from the selected clusters (class divisions) were included in the study, resulting in a final sample of 823 participants, which was used for the present analysis. Although the sample size was originally calculated for the parent study assessing eating disorder risk, the final sample of 823 participants provided adequate precision for the present analysis. Based on the observed survey-weighted SMA prevalence of 38.07%, the achieved sample size yielded a margin of error of approximately ± 3.3% points at the 95% confidence level, indicating adequate precision for estimating the prevalence of SMA. For the main exposure variable, daily social media duration, the achieved sample provided approximately 99.3% power at a two-sided 5% significance level to detect the observed difference in SMA prevalence between students using social media for > 5 h/day and those using it for 0–5 h/day. However, the study was not specifically designed or powered to detect all adjusted associations or subgroup differences; therefore, findings from the multivariable analyses should be considered exploratory and interpreted with appropriate caution.

### Sampling technique

Participants were recruited using a multi-stage cluster sampling technique, selecting two districts (Kollam and Kozhikode) from Kerala’s northern and southern regions. Four colleges per district (two Arts & Science and two Engineering, from government/aided and private sectors) were randomly chosen, followed by simple random sampling of classrooms. Detailed sampling methodology has been previously published [15]. Prior permission to conduct the study was obtained from the principals of the selected colleges. The principals were initially contacted by telephone, followed by email communication explaining the study objectives, procedures, and ethical safeguards. All selected colleges agreed to participate after receiving information regarding the study objectives, procedures, and ethical safeguards. Formal institutional permission was obtained from each participating college through official communication. Faculty representatives facilitated only logistical arrangements such as identifying classrooms and scheduling data collection sessions. They were not involved in inviting students to participate, obtaining consent, or collecting completed questionnaires.

The study was presented as an optional research activity rather than a mandatory academic exercise. Faculty members were not present during questionnaire administration. Before data collection, eligible students were provided with a participant information sheet and a written informed consent form explaining the purpose of the study, the voluntary nature of participation, the confidentiality of responses, the absence of incentives, and the right to decline participation or leave any question unanswered without penalty. Questionnaires were distributed individually to eligible students. Students who did not wish to participate could decline participation, return a blank questionnaire, or leave items unanswered without providing any explanation. Participation status was not disclosed to faculty members or college authorities. Students were informed that non-participation would not affect their academic standing, attendance, grades, or relationship with faculty members. Only students present in the selected classrooms on the scheduled day of data collection were approached. Students absent on that day were not contacted or followed up separately.

### Study tools

A validated self-report questionnaire, including sociodemographic characteristics, social media usage patterns, and the BSMAS, was used. BSMAS, a six-item self-report instrument developed by Andreassen et al., which measures salience, mood modification, tolerance, withdrawal, conflict, and relapse related to social media use [3]. Each item is scored on a five-point Likert scale from 1 (very rarely) to 5 (very often), yielding a total score of 6 to 30. BSMAS has been used in previously conducted studies in India [16]. The instrument is copyrighted but may be used for academic research with appropriate citation.

Consistent with previous literature using the BSMAS, a score of ≥ 19 indicated SMA, and ≥ 24 indicated social media disorder (SMD). The ≥ 19 threshold has also been applied in Indian studies using the BSMAS among young adults [17]. However, these cut-offs have not undergone formal cultural validation for the Indian population. The term “social media addiction” in this study refers to a screening-based construct reflecting problematic and excessive social media use identified using the BSMAS and does not represent a clinical psychiatric diagnosis.

### Independent and outcome variables

The independent variables were age, gender, marital status, social class (based on revised BG Prasad’s classification) [18], type of college, course pursued, and social media usage characteristics. Daily social media duration was defined as the self-reported average time spent on social media per day over the preceding week. Variables included in the multivariable analysis were selected using a conceptually informed approach based on prior literature and study objectives rather than solely on statistical significance. The outcome variable was SMA assessed using the BSMAS.

### Statistical analysis

The collected data were entered into EpiData Version 4, and the statistical analyses were done using STATA Version 14. Survey analyses accounted for the multistage sampling design by specifying colleges as the primary sampling units, districts as strata, and applying normalized sampling weights. The prevalence of SMA and SMD was estimated with 95% confidence intervals (CIs). Associations between independent variables and SMA were initially assessed using the Rao-Scott adjusted Chi-square test and survey-weighted unadjusted prevalence ratios (uPRs) with 95% CIs estimated using generalized linear models (GLM) with a Poisson family and log link. Multivariable survey-weighted GLM with a Poisson distribution and log link was performed to estimate adjusted prevalence ratios (aPRs) while simultaneously controlling for potential confounding variables. The multivariable model was developed using a conceptually informed approach rather than relying solely on statistical significance in the bivariate analysis. Age and gender were retained a priori, and variables with plausible relevance to SMA based on prior literature and study objectives, including course, social class, marital status, most used social media platform, and daily social media duration, were included in the adjusted model. Survey-weighted generalized linear models with a Poisson family and log link were used to estimate adjusted prevalence ratios (aPRs) with 95% CIs. Poisson regression with robust survey variance estimation was chosen because it provides direct and interpretable estimates of prevalence ratios for common outcomes in cross-sectional studies. Daily social media duration and frequency of social media access were moderately correlated (Spearman’s rho = 0.401, *p* < 0.001). As both variables represented closely related aspects of social media engagement, daily duration was retained in the multivariable model because it is more directly interpretable and has been more commonly reported in previous studies. The relationship between daily social media duration and BSMAS score was further examined using a scatter plot and Spearman’s rank correlation coefficient. A *p*-value < 0.05 was considered statistically significant.

## Results

A total of 823 undergraduate students participated in the study. All students present in the selected classrooms consented to participate and completed the questionnaire. No incentives were provided. The survey-weighted mean age of the participants was 19.76 years (SE = 0.14). Females constituted 63.9% of the weighted study population, while 49.0% were from private colleges. Approximately two-thirds (64.1%) were enrolled in Arts and Science courses, and participants were distributed between Kozhikode (53.2%) and Kollam (46.8%). About 56.8% belonged to social class I or II, and the majority resided at home (77.1%). Most participants were unmarried (97.4%). Based on body mass index, 55.2% had normal BMI, 30.2% were underweight, and 14.7% were overweight or obese (Table 1).

**Table 1:** Sociodemographic characteristics of study participants (*N* = 823)

Variable	Category	*n*	Weighted %
Age (years)	Weighted Mean (SE)	—	19.76 (0.14)
Gender	Male	327	36.1
	Female	496	63.9
Type of college	Government	310	35.2
	Aided	99	15.8
	Private	414	49
Course	B.Tech	403	35.9
	Arts and Science	420	64.1
District	Kozhikode	408	53.2
	Kollam	415	46.8
Social class*	Class I (≥₹9,098)	251	28.6
	Class II (₹4,549-9,097)	235	28.2
	Class III (₹2,729-4,550)	142	16.8
	Class IV (₹1,365-2,728)	147	19.1
	Class V (<₹1,365)	48	7.4
Place of stay	Home	607	77.1
	Hostel	158	16.8
	Paying guest	58	6
Marital status	Married	18	2.6
	Unmarried	805	97.4
Body mass index (BMI)^†^	Underweight (< 18.5 kg/m²)	233	30.2
	Normal (18.5–24.9 kg/m²)	465	55.2
	Overweight/Obese (≥ 25 kg/m²)	125	14.7

*Based on Modified BG Prasad classification, October 2023 (Rupees/month)

^†^Based on self-reported height and weight, Weighted percentages were estimated using survey weights accounting for the sampling design.﻿

Instagram was the most commonly used social media platform (60.1%), followed by YouTube (26.3%). The median daily duration of social media use was 330 min (IQR: 220–450). More than one-third of participants (37.0%) accessed social media 11–20 times per day, while 32.3% reported accessing it more than 20 times daily. Interest in food-related social media content was reported by 76.6% of participants, and 56.5% used social media for fitness-related purposes (Table 2).

**Table 2:** Social media usage characteristics of study participants (*N* = 823)

Variable	Category	*n*	Weighted %
Most used social media platform	Instagram	497	60.1
	YouTube	216	26.3
	Others	110	13.6
Daily social media duration (minutes)*	Median (IQR)	—	330 (220–450)
Frequency of social media access (times/day)^†^	≤ 10 times	245	30.8
	11–20 times	311	37
	> 20 times	267	32.3
Interest in food-related social media content	Yes	620	76.6
	No	203	23.4
Use of social media for fitness purposes	Yes	473	56.5
	No	350	43.5

*Self-reported average daily use over the preceding week

^†^Self-reported daily frequency of social media access, Weighted percentages were estimated using survey weights accounting for the sampling design

Table 3 presents the distribution of responses to the BSMAS questionnaire. Most participants reported behaviours related to social media use at least “sometimes.” The most frequently reported behaviours were spending a lot of time thinking about social media (51.2%) and unsuccessfully trying to reduce social media use (51.3%). Nearly half (49.1%) reported feeling an increasing urge to use social media more and more, while 44.0% reported using social media to cope with personal problems. Approximately one-third (33.2%) reported feeling restless or troubled when unable to use social media, and 38.8% indicated that excessive social media use had negatively affected their studies at least sometimes.

**Table 3:** Distribution of responses to the Bergen Social Media Addiction Scale (BSMAS) questionnaire (*N* = 823)

BSMAS questions	Very Rarely *n* (Weighted %)	Rarely *n* (Weighted %)	Sometimes *n* (Weighted %)	Often *n* (Weighted %)	Very often *n* (Weighted %)
Item 1 - “…spent a lot of time thinking about social media”	110 (12.8)	211 (24.0)	399 (51.2)	79 (9.1)	24 (2.9)
Item 2 - “…felt an urge to use social media more and more”	48 (5.7)	145 (16.6)	406 (49.1)	169 (22.7)	55 (5.9)
Item 3 - “…used social media to forget about personal problems”	93 (10.8)	135 (16.2)	358 (44.0)	161 (19.9)	76 (9.1)
Item 4 - “…tried to cut down on the use of social media without success”	76 (9.0)	157 (19.2)	423 (51.3)	126 (15.4)	41 (5.1)
Item 5 - “…become restless or troubled if you have been prohibited from using social media”	203 (23.8)	204 (25.0)	265 (33.2)	113 (13.2)	38 (4.8)
Item 6 - “…used social media so much that it has had a negative impact on your job/studies”	101 (12.5)	163 (18.2)	329 (38.8)	145 (20.0)	85 (10.5)

**Fig. 1:**
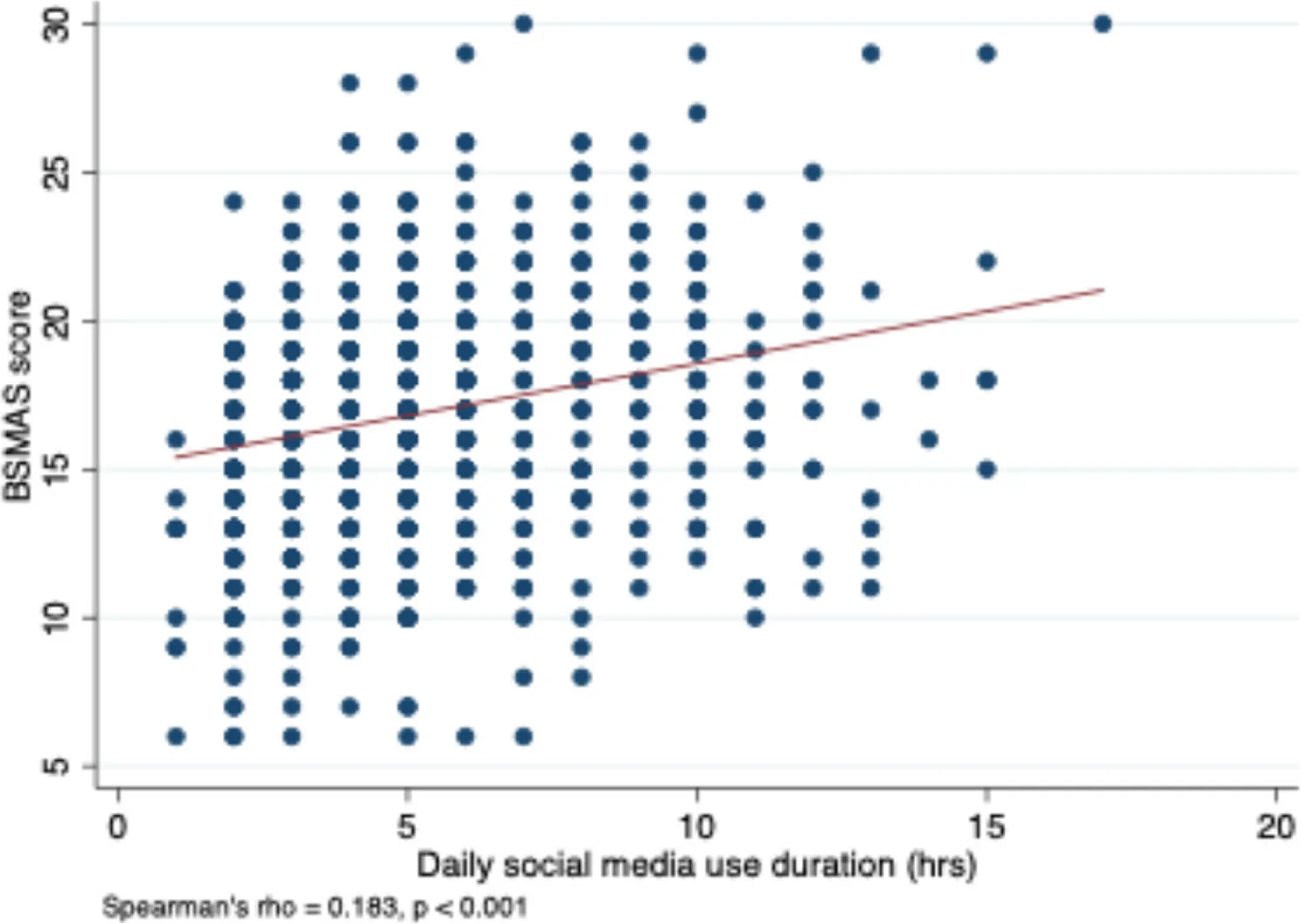
Relationship between daily social media duration and BSMAS score

The survey-weighted prevalence of SMA was 38.07% (95% CI: 33.56–42.81), while the prevalence of SMD was 4.03% (95% CI: 2.69–5.99). Among participants with SMA, 12.67% met the criteria for SMD. Table 4 presents the factors associated with SMA. In the multivariable survey-weighted analysis, female students had a 42% higher prevalence of SMA than male students (APR = 1.42; 95% CI: 1.07–1.87; *p* = 0.018). Married students also had a higher prevalence of SMA than unmarried students (APR = 1.73; 95% CI: 1.33–2.24; *p* < 0.001), although this finding should be interpreted cautiously because only 18 participants were married. Students reporting more than five hours of daily social media use had a 41% higher prevalence of SMA than those using social media for 0–5 h per day (APR = 1.41; 95% CI: 1.12–1.78; *p* = 0.006). Age, course, social class, and most-used social media platform were not independently associated with SMA after adjustment. A weak but statistically significant positive correlation was observed between daily social media duration and BSMAS score (Spearman’s rho = 0.183, *p* < 0.001) (Fig. 1).

**Table 4:** Factors associated with Social Media Addiction (SMA) (*N* = 823)

Category	*n*	SMA *n* (weighted %)	No SMA *n* (weighted %)	UPR (95% CI)	*P* value	APR (95% CI)	*P* value
Age (years)							
18	148	59 (41.5)	89 (58.5)	1.43 (1.06–1.95)	0.023	1.30 (0.95–1.79)	0.098
19	204	82 (42.8)	122 (57.3)	1.48 (0.99–2.20)	0.054	1.45 (0.97–2.16)	0.066
20	225	81 (39.6)	144 (60.4)	1.37 (0.91–2.05)	0.119	1.35 (0.90–2.04)	0.134
21	114	34 (28.9)	80 (71.1)	1	-	1	-
22	132	44 (30.8)	88 (69.2)	1.06 (0.61–1.86)	0.814	0.98 (0.55–1.74)	0.950
Gender							
Male	327	101 (30.7)	226 (69.4)	1	-	1	-
Female	496	199 (42.3)	297 (57.7)	1.38 (1.10–1.74)	0.010	1.42 (1.07–1.87)	**0.018**
Course							
B.Tech	403	138 (33.7)	265 (66.3)	1	-	1	-
Arts & Science	420	162 (40.5)	258 (59.5)	1.20 (1.03–1.41)	0.025	1.07 (0.90–1.28)	0.403
Social class							
Class I	251	106 (45.8)	145 (54.2)	1.40 (1.10–1.80)	0.011	1.23 (0.99–1.53)	0.061
Class II	235	89 (38.6)	146 (61.4)	1.18 (0.97–1.44)	0.088	1.11 (0.88–1.41)	0.355
Class III and above	337	105 (32.6)	232 (67.4)	1	-	1	-
Place of stay							
Home	607	221 (38.2)	386 (61.8)	1.39 (0.91–2.10)	0.116	-	-
Hostel	158	62 (41.1)	96 (58.9)	1.49 (0.74–2.98)	0.240	-	-
Paying guest	58	17 (27.6)	41 (72.4)	1	-	-	-
Marital status							
Married	18	12 (71.7)	6 (28.3)	1.93 (1.53–2.43)	< 0.001	1.73 (1.33–2.24)	**< 0.001**
Unmarried	805	288 (37.2)	517 (62.8)	1	-	1	-
BMI							
Underweight	233	87 (40.6)	146 (59.4)	1.16 (0.90–1.49)	0.223	-	-
Normal	465	161 (35.0)	304 (65.0)	1	-	-	-
Overweight/obese	125	52 (44.5)	73 (55.5)	1.27 (0.84–1.92)	0.235	-	-
Most used social media							
Instagram	497	196 (40.9)	301 (59.1)	1.27 (0.69–2.35)	0.409	1.24 (0.65–2.36)	0.492
YouTube	216	73 (34.7)	143 (65.3)	1.08 (0.73–1.61)	0.680	1.02 (0.63–1.66)	0.936
Others	110	31 (32.1)	79 (67.9)	1	-	1	-
Social media duration*							
> 5 h	460	197 (46.9)	263 (53.1)	1.47 (1.21–1.79)	0.001	1.41 (1.12–1.78)	**0.006**
0–5 h	363	103 (31.8)	260 (68.2)	1	-	1	-
Type of college							
Government	310	107 (37.5)	203 (62.5)	1	-	-	-
Aided	99	35 (38.5)	64 (61.5)	1.03 (0.71–1.49)	0.878	-	-
Private	414	158 (38.4)	256 (61.6)	1.02 (0.72–1.45)	0.889	-	-

*Self-reported average time spent on social media per day over the preceding week

## Discussion

The prevalence of SMA among undergraduate students in this study was 38.07%. Among them, SMD prevalence was 4.03%. These findings are similar to those of a previous study by Ramesh et al. among adolescents in the neighbouring state of Karnataka, although the authors used a different scale. Our findings were slightly higher than the 31% SMA rate reported by Cheng et al.’s meta-analysis [7]. The relatively high prevalence of SMA observed in this study may also be understood in the context of Kerala’s unique socio-digital environment. Kerala has one of the highest levels of internet penetration, smartphone access, and digital literacy in India, facilitating early and sustained exposure to social media platforms among young adults [13, 19, 20]. In addition, widespread availability of affordable internet services and high engagement with online content for educational, social, and lifestyle purposes may contribute to increased screen time and habitual use [21]. Kerala’s high internet penetration and digital access may increase opportunities for prolonged engagement with social media platforms, which in turn could contribute to SMA among vulnerable individuals [11, 22]. These findings highlight the need for context-specific digital health interventions in Kerala, where high connectivity, while beneficial, may also increase vulnerability to problematic use. Symptom-level responses from the BSMAS indicated that cognitive preoccupation and craving-related behaviours were commonly reported among participants. Frequent thoughts about social media use and urges to engage more may reflect early behavioural features associated with SMA.

In the current study, three variables were found to be significantly associated with SMA in adjusted analyses: gender, duration of social media use and marital status. Differences in social media usage patterns and engagement styles may partly explain the higher prevalence observed among female participants. Prior research suggests that females are more likely to use social networking platforms for social interaction, emotional expression, and relationship maintenance, which may increase vulnerability to excessive engagement [23, 24]. In addition, platform features such as continuous scrolling, personalised content feeds, and social feedback mechanisms (e.g., likes and comments) may further enhance engagement and reinforce usage patterns [25]. Together, these factors indicate that both behavioural exposure (duration of use) and socio-behavioural differences play an important role in the development of SMA among undergraduate students.

Duration of social media use emerged as the strongest independent factor associated with SMA, highlighting prolonged screen time as a potentially modifiable behavioural target for intervention. The median daily social media use of 330 min observed in the present study was considerably higher than the global average of 151 min per day reported in 2023 [7], suggesting that undergraduate students from the selected districts of Kerala may have substantially greater exposure to social media than the global population, which could contribute to a higher burden of social media addiction. Students who reported using social media for more than five hours daily had 1.41 times higher prevalence of developing SMA than those who used it less. A statistically significant but weak positive correlation was observed between duration of social media use and BSMAS scores, suggesting that duration alone explains only part of the variation in social media use behaviours. This is consistent with Awasthi et al. [22], who found that prolonged online activity was significantly associated with sleep disturbances, depressive symptoms, and reduced academic performance among medical students. Their work suggests that excessive digital engagement may contribute to a detrimental cycle of compulsive behaviour and psychological burden. Prolonged social media use may contribute to behavioural and psychological changes, while pre-existing emotional or behavioural vulnerabilities may also increase susceptibility to SMA.

In the adjusted analysis, marital status emerged as a significant factor associated with SMA, with married students demonstrating a higher prevalence of SMA compared to unmarried students. This finding should be interpreted cautiously because only a small proportion of participants were married. Nevertheless, married students may experience additional social, academic, and family responsibilities that may increase reliance on social media platforms for communication, emotional support, or stress relief [26, 27]. Given the limited evidence regarding the relationship between marital status and SMA among undergraduate populations, further studies with larger and more diverse samples are needed to determine whether this association is reproducible or reflects residual confounding.

The strengths of this study include a large and diverse sample of undergraduate students representing different academic streams and institutional settings from the selected districts of Kerala. However, several limitations should be considered. First, the cross-sectional design precludes conclusions regarding temporal or causal relationships between the identified factors and SMA. Furthermore, the study was not specifically designed or powered to investigate factors associated with SMA, and therefore, the observed associations should be interpreted as exploratory. Although multivariable analysis was used to adjust for measured covariates, residual confounding due to unmeasured factors, such as psychological characteristics, academic stress, personality traits, or digital literacy, cannot be excluded. In addition, the study was not explicitly guided by a behavioural theory, and future studies may benefit from theory-informed approaches to strengthen causal inference and variable selection.

The study was conducted among undergraduate students from two districts of Kerala, which may limit the generalisability of the findings to students from other regions or educational settings. Students who were absent during data collection were not included, introducing the possibility of selection bias. Although participation was voluntary and several measures were implemented to minimise coercion, the absence of refusals among students approached may reflect residual participation pressure, social desirability bias, or cultural norms favouring participation in academic activities, all of which are difficult to eliminate completely in classroom-based surveys. In addition, social media use and related behaviours were assessed using self-reported measures and may therefore be subject to recall and social desirability bias.

An important limitation relates to the use of internationally recommended BSMAS cut-offs, which have not yet been formally validated for the Indian population. Consequently, the reported prevalence of SMA and SMD should be interpreted as the proportion of students meeting a screening threshold rather than the prevalence of clinically diagnosed addiction. Cultural differences in social media use patterns and response behaviour may influence the performance of these cut-offs and, therefore, the estimated prevalence reported in this study. Future research should validate the BSMAS and establish culturally appropriate cut-offs for Indian young adults to improve the accuracy and interpretability of prevalence estimates.

## Conclusion

About 38% of undergraduate students from the selected districts of Kerala were found to have SMA, with 4% reporting SMD. Female students and those spending more than five hours daily on social media had a higher prevalence of SMA. These findings underscore the need for targeted digital well-being interventions in higher education settings, particularly to reduce excessive screen time and promote healthy online behaviours. Colleges may consider implementing structured awareness programs, screen-time monitoring initiatives, and counselling support to improve digital self-regulation and coping mechanisms. Such context-specific strategies are essential to mitigate the growing burden of social SMA among young adults. 

## Data Availability

The materials and datasets generated and analysed during the current study are available from the corresponding author on reasonable request.
